# Supplementary material for: Interferon inducible X-linked gene CXorf21 may contribute to sexual dimorphism in Systemic Lupus Erythematosus
Source: Nat Commun. 2019 May 15;10:2164. doi: 10.1038/s41467-019-10106-2 (PMC6520347; doi:10.1038/s41467-019-10106-2)
Supplement: Supplementary file 1 — Supplementary Information [file 41467_2019_10106_MOESM1_ESM.pdf]

Supplementary Information

Interferon inducible X-linked gene *CXorf21* may contribute to sexual dimorphism in Systemic Lupus Erythematosus

Christopher A. Odhams, Amy L. Roberts, et al.

Supplementary Figure 1: Flow diagram of resources, databases, experiments, and analyses performed in manuscript. Each numbered box represents a subheading within the results section in the manuscript. Per box, each dataset (either publicly available or generated in house) is listed and referenced. The subsequent subheadings denote the type of analysis conducted and the statistical methodology used (if applicable).

1

Genetic refinement of the Xp21.2 SLE susceptibly locus

1. SLE GWAS in European individuals (*Bentham and Morris et al 2015 Nat Genet.* 26502338)
   - 1.1. Imputation across Xp21.2 to UK10K-1000 Genomes Project Phase 3 reference panel (IMPUTE2)
   - 1.2. Case-control association test (SNPTEST logistic regression)
   - 1.3. Conditional analysis (SNPTEST with genotype covariate)
   - 1.4. Haplotype association analysis (Haploview chi-square test)
   - 1.5. Inactivation association model (SNPTEST likelihood ratio test)

2

The rs887369 risk haplotype increases the expression of *CXorf21* in male and female LCLs

1. Geuvadis RNA-Seq genotype-expression cohort in European LCLs (*Lappalainen et al 2013 Nature* 24037378)
   - 1.1. *cis*-eQTL analysis in males at the Xp21.2 locus (linear regression)
   - 1.2. *cis*-eQTL analysis in females at the Xp21.2 locus (linear regression)
2. TwinsUK RNA-Seq genotype-expression cohort in European LCLs (*Buil et al 2015 Nat Genet.* 25436857)
   - 2.1. *cis*-eQTL analysis in non-skewed females at the Xp21.2 locus (linear regression)
3. *CXorf21* mRNA (qPCR) and protein-level (Western Blot) quantification in female LCLs (in house experiment)
   - 3.1. Verification of rs887369 - *CXorf21 cis*-eQTL effect at Xp21.2

3

The rs887369 risk haplotype increases expression of *CXorf21* in activated immune cells

1. Microarray genotype-expression cohort in European *ex vivo* immune cells (*Fairfax et al 2014 Science* 24604202)
   - 1.1. *cis*-eQTL analysis in males at the Xp21.2 locus (linear regression)

4

Epigenetic fine-mapping of the associated haplotype

1. Roadmap Epigenomics Project (*Roadmap Epigenomics Consortium et al 2015 Nature* 25693563)
   - 1.1. Epigenetic fine-mapping in 127 cell and tissue types across 12 histone marks (ChIP-Seq)
2. Blueprint Epigenome consortium (*Martens et al 2013 Haematologica* 24091925)
   - 2.1. Epigenetic fine-mapping in 24 cell and tissue types across 8 histone marks (ChIP-Seq)
3. miRDB database for predicted microRNA targets in animals (*Wong et al 2015 Nucleic Acids Res.* 25378301)
   - 3.1. Analysis of microRNA binding site disruption in 3'-UTR of *CXorf21* (http://mirdb.org/index.html)

5

The SLE-associated haplotype interacts with the promoter of *CXorf21*

1. Promoter capture Hi-C data from primary immune cell-types (*Schofield et al 2016 Bioinformatics* 27153610)
   - 1.1. Assessment of chromatin interaction across the Xp21.2 associated locus (CHiCP resource)
2. Roadmap Epigenomics Project (*Roadmap Epigenomics Consortium et al 2015 Nature* 25693563)
   - 2.1. Correlation between Hi-C interaction score and chromatin mark signal enrichment
3. Transcription Factor ChIP-Seq Uniform Peaks from ENCODE/Analysis (*ENCODE Project Consortium*)
   - 3.1. Assessment of RNA polymerase II binding at the Xp21.2 associated locus across different cell types

6

Sexual dimorphic expression of *CXorf21* is magnified in activated immune cells

1. The Genotype-Tissue Expression (GTEx) project (*The GTEx Consortium Nat. Genet. 2013* 23715323)
   1.1. Assessment of sexual dimorphic expression across cell and tissue types (unpaired *t*-test)
2. *CXorf21* protein-level (Western Blot) quantification in male and female LCLs (in house experiment)
   2.1. Assessment of sexual dimorphic expression at protein-level in LCLs (unpaired *t*-test)
3. Microarray genotype-expression cohort in European *ex vivo* immune cells (*Fairfax et al 2014 Science* 24604202)
   3.1. Assessment of sexual dimorphic expression in *ex vivo* resting & stimulated immune cells (unpaired *t*-test)

7

***CXorf21* is a likely interferon response gene**

1. Microarray cohort of resting and IFN-α *ex vivo* B-cells in females (in house experiment)
   1.1. Assessment of genes up-regulated in B-cells in response to IFN-α (fitted ANOVA model)
2. Transcription Factor ChIP-Seq Uniform Peaks from ENCODE/Analysis (*ENCODE Project Consortium*)
   2.1. Identification of IFN regulators at the *CXorf21* promoter in LCLs (ChIP-Seq)

8

Functional characterisation of *CXorf21* at the Xp21.2 SLE susceptibly locus

1. The Human Protein Atlas (*Pontén et al 2008 J Pathol. 2008* 18853439)
   1.1. Identification of *CXorf21* cell and tissue types expression at protein-level
2. Roadmap Epigenomics Project (*Roadmap Epigenomics Consortium et al 2015 Nature* 25693563)
   2.1. Assessment of chromatin landscape within the Xp21.2 associated locus (ChIP-Seq
3. Blueprint Epigenome consortium (*Martens et al 2013 Haematologica* 24091925)
   3.1. Identification of *CXorf21* cell and tissue types expression at mRNA-level (RNA-Seq)
4. BioGPS (*Wu et al Genome Biol 2009* 19919682)
   4.1. Identification of *CXorf21* cell and tissue types expression at mRNA-level (microarray)
5. COXPRESdb (*Okamura et al Nucleic Acids Res. 2015* 25392420)
   5.1. Co-expression analysis of CXorf21 across human cell and tissue-types (COXPRES algorithm)
6. BioPlex (*Huttlin et al Nature 2017* 28514442)
   6.1. Assessment of *Cxorf21* protein-protein interaction (affinity-purification mass spectrometry)

9

CXORF21 protein abundance is correlated with SLEDAI in young females

1. CXORF21 protein-level (flow cytometry) quantification in CD14+ and CD19+ *ex vivo* cells (in house experiment)
   1.1. Comparison of abundance between SLE patients and healthy controls (unpaired t-test)
   1.2. Correlation between protein abundance and disease activity (Linear regression)

10

CXORF21 protein may act within endosomal pathway

1. Multispectral imaging flow cytometry across various organelles in PBMCs and GM12878 (in house experiment)
   1.1 Co-localisation of CXORF21 protein with nuclear, Golgi, and lysosome (IDEAS software algorithm)
2. Structured Illumination Microscopy (SIM) in resting and stimulated *ex vivo* B cells (in house experiment)
   2.1. Co-localisation of CXORF21 protein with TLR7 and LC3 (Pearson and Mander's)
3. CXORF21 protein-level (Western Blot) quantification in starved LCL (in house experiment)
   3.1. Assessment of CXORF21 protein as autophagy substrate in starved LCL

Supplementary Figure 2: *Cis*-eQTL analysis of SLE associated SNP, rs887369, on gene expression across an array of immune cell-types in females only, with qPCR validation. **a:** Using the Geuvadis RNA-Seq cohort[1] in females in <u>lymphoblastoid cell lines</u>. *CXorf21*, *GK*, and *TAB3* were included as had RPKM > 1; the remaining genes were dropped from quality control. **b:** *in vitro* qPCR validation of effect seen in <u>lymphoblastoid cell lines</u>, using 5 homozygote risk rs887369 [CC] individuals against 5 homozygotes non-risk rs887369 [AA] individuals (see methods). **c:** Immunoblot of allelic effect on rs887369 on CXORF21 protein abundance in females (raw data shown in Source Data) in <u>lymphoblastoid cell lines</u>. **d:** *cis*-eQTL analysis of rs887369 against *CXorf21* expression using an array of <u>primary *ex vivo* immune cell types</u> from the *Fairfax et al*[2] and *Naranbhai et al*[3] cohorts in females. Box-plots show minimum (Q1-1.5*IQR), 25th percentile (Q1), Median, 75th percentile (Q3), and maximum Q3+1.5*IQR. Source data are provided as a Source Data file.

Figure S2

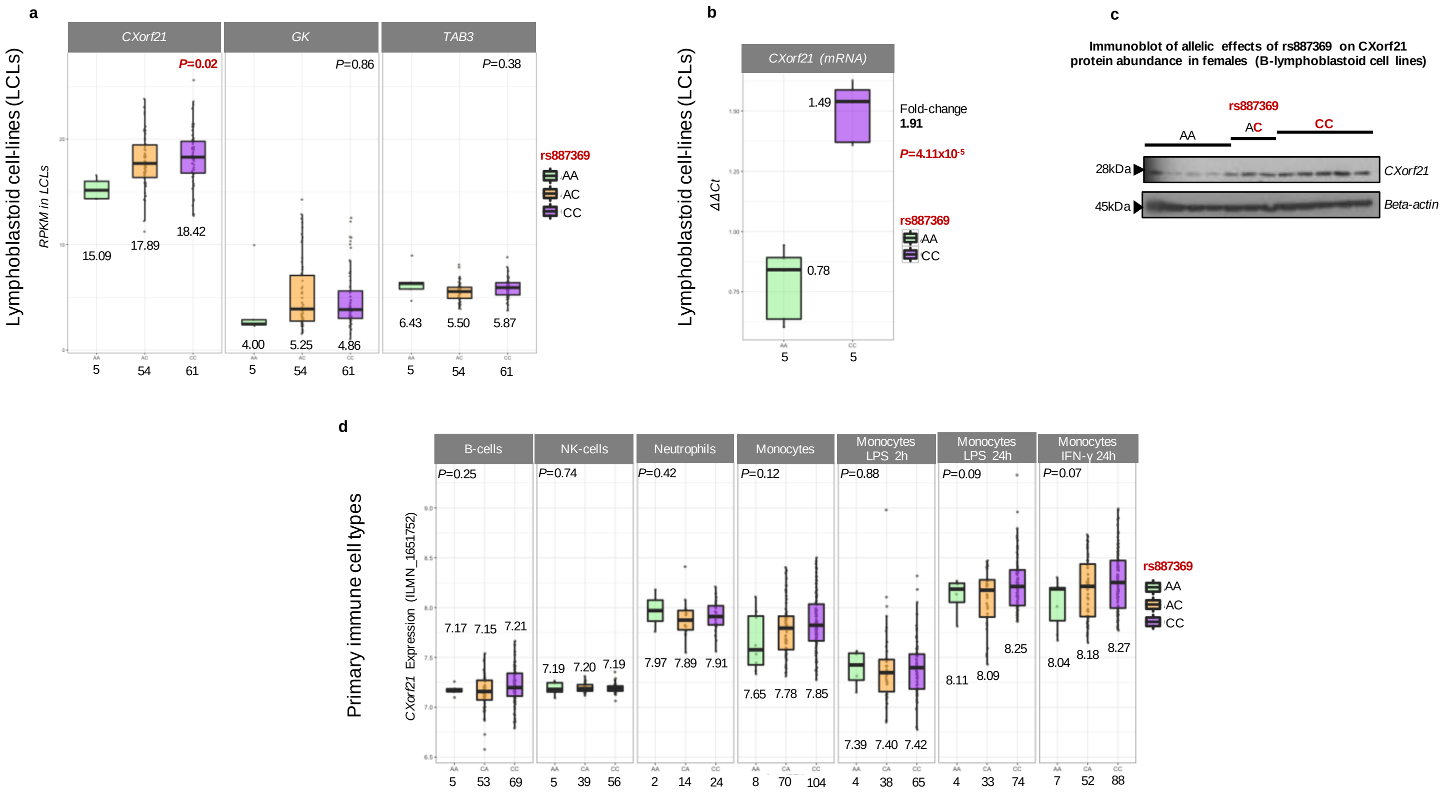

Supplementary Figure 3: Validation of antibody used to target the CXORF21 protein. In duplicate, the GM12878 cell line (LCL) was subject to siRNA mediated knockdown of *CXorf21* (see methods). A 44% knockdown of CXORF21 was achieved at 48hours post-transfection – assessed by quantitative Western Blot using ImageJ. Source data are provided as a Source Data file.

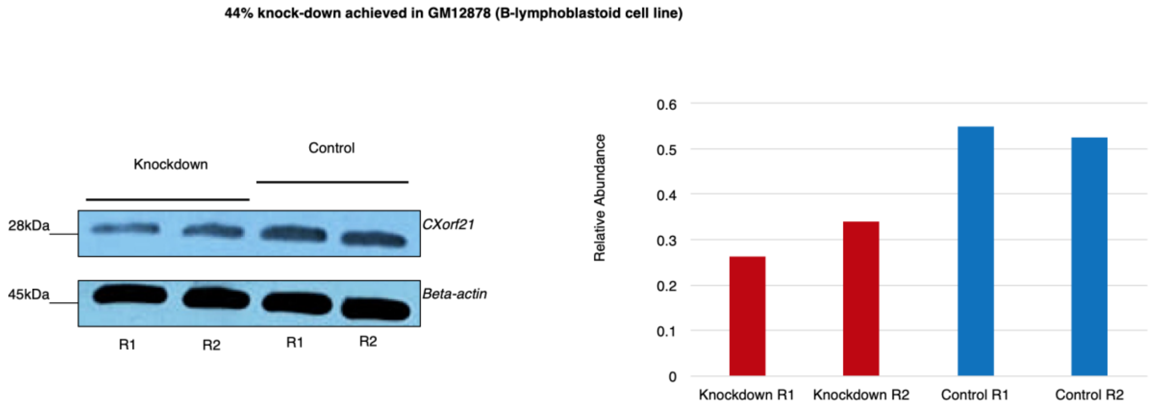

Supplementary Figure 4: Differential H3K36me3 signal between the male and female samples across cell-types using the Blueprint Data[4]. Box-plots show minimum (Q1-1.5*IQR), 25th percentile (Q1), Median, 75th percentile (Q3), and maximum Q3+1.5*IQR.

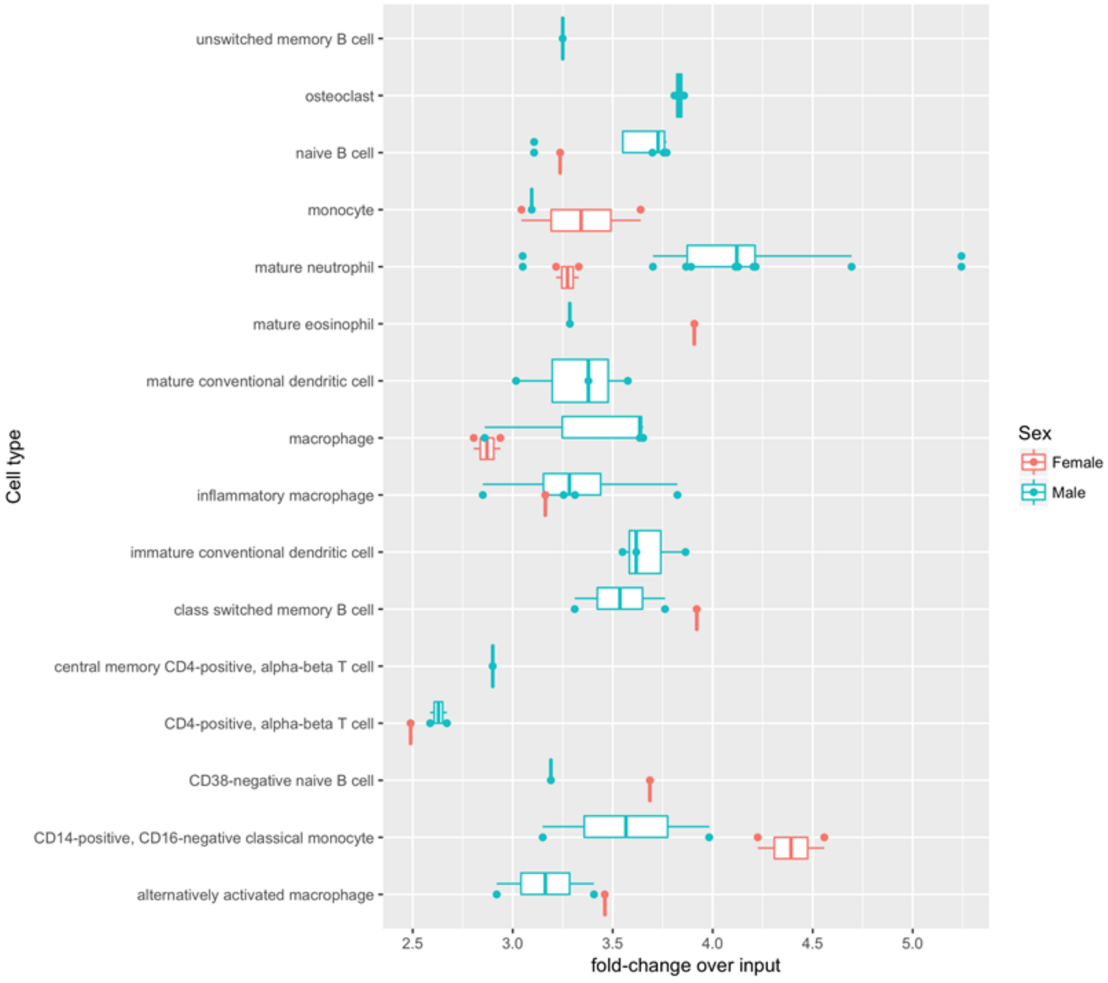

Supplementary Figure 5: Transcription factor and RNA polymerase subunit binding in LCLs (GM12878) across the *CXorf21* locus taken from the ENCODE project[5]. The associated SNPs are shown in red. The 3′ associated region was found to interact with the *CXorf21* promoter (see results). Multiple immune-regulatory transcription factors were found to bind to the promoter of *CXorf21* including interferon regulators. The RNA polymerase 2 subunit (POLR2A) was found to bind around the associated region suggesting the looping interaction between the 3′ associated region of CXorf21 and its promoter is necessary for active gene transcription of *CXorf21*.

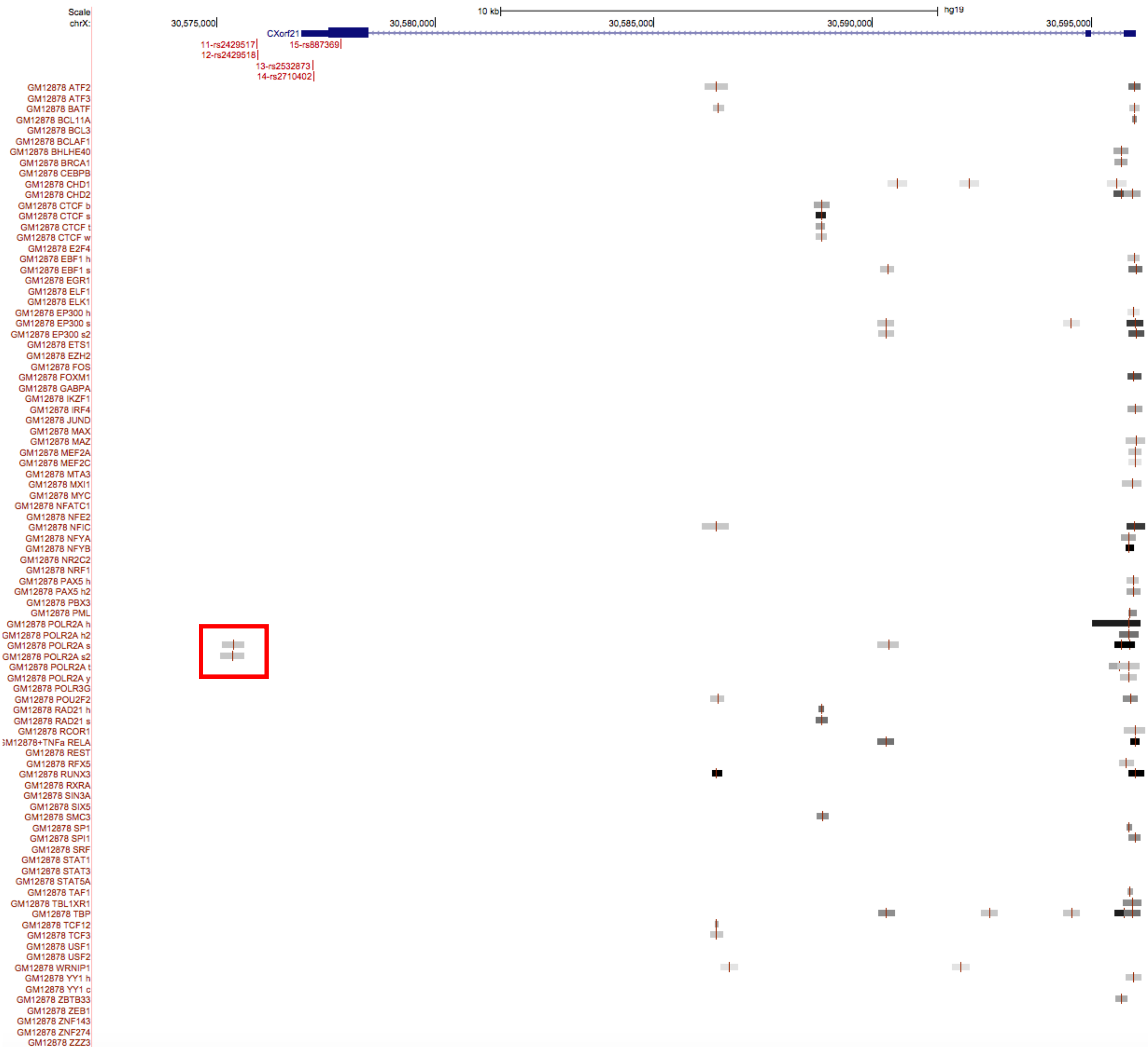

Supplementary Figure 6: Sex-dependent gene and protein expression profile of *CXorf21* in lymphoblastoid cell lines (LCLs) and *ex vivo* primary immune cell types. **a:** RNA-Seq expression of *CXorf21* in LCLs using the GTEx project data[6]. In red are females (RPKM=25.812, n=43) and in blue are males (RPKM=14.350, n=75); suggesting potential escape from X-inactivation of *CXorf21* in females (fold-change: 1.78). **b:** Validation of sex-dependent effect of *CXorf21* measured at protein level by Western Blot. The lysates of four male and four female LCL cell lines (resting) were assessed; where a fold-change of 3.6 was observed (Beta-actin used as loading control). **c**: Expression profile of *CXorf21* in primary *ex vivo* cell types between male and female individuals using separate studies by *Fairfax et al*[2] and *Naranbhai*[3] *et al* (see methods). X-axis shows number of individuals per sample; Y-axis is the log_2 normalised relative expression of *CXorf21* measured by microarray (probe ID: ILMN_1651752); the mean of each sample is shown and the *P*-value of *t*-test between sexes (see methods). Box-plots show minimum (Q1-1.5*IQR), 25th percentile (Q1), Median, 75th percentile (Q3), and maximum Q3+1.5*IQR.

Figure S6

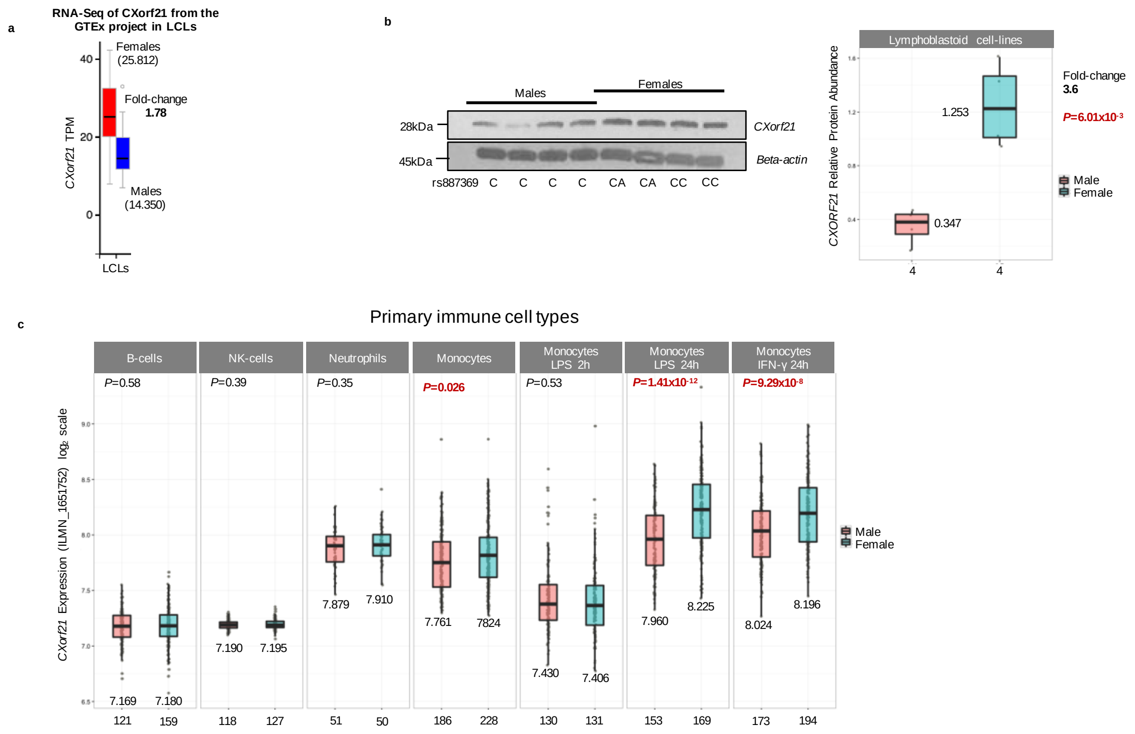

Supplementary Figure 7: Tissue/cell-type expression profiles of the eight genes within the 1Mb locus of lead SLE associated SNP rs887369 (chrX: 30,077,846-31,077,845) taken from The Human Protein Atlas[7] RNA-Sequencing data. RNA-Seq tissue/cell data are reported as mean TPM (transcripts per million), corresponding to mean values of the different individual samples from each tissue. Color-coding is based on tissue groups, each consisting of tissues with functional features in common. Heat colour of expression is relative to each gene individually.

Tissue Type	Sample	*MAGEB2*	*MAGEB3*	*MAGEB4*	*MAGEB1*	*NR0B1*	*CXorf21*	*GK*	*TAB3*
Neuronal	Cerebral cortex	0	0	0	0	1.4	2.2	10.4	15.3
Endocrine	Adrenal gland	0	0	0	0	27.9	2.5	13.1	10.1
	Thyroid gland	0	0	0	0	0	1.1	11	16.7
	Parathyroid gland	0	0	0	0	0	1.4	16	52.6
Immune system	**Lymph node**	0	0	0	0	0	**11.9**	12.4	10.5
	**Bone marrow**	0	0	0	0	0	**12**	4.3	10.6
	**Appendix**	0	0	0	0	0	**11.8**	10.8	11.1
	**Spleen**	0	0	0	0	0	**13.4**	11.4	11.8
	**Tonsil**	0	0	0	0	0	**9.4**	13.9	12
Muscle	Skeletal muscle	0	0	0	0	0	0	0	3.7
	Heart muscle	0	0	0	0	0	0	2.6	5.8
	Smooth muscle	0	0	0	0	0	3	6.4	19.6
Lung	Lung	0	0	0	0	0	6.7	24.6	13.5
Liver	Gallbladder	0	0	0	0	0	6	38.6	12.9
	Liver	0	0	0	0	0	1.2	41.6	13.9
Pancreas	Pancreas	0	0	0	0	0	0	0	5.2
Digestive	Salivary gland	0	0	0	0	0	0	1.8	3.8
	Duodenum	0	0	0	0	0	4.8	89.7	8
	Small intestine	0	0	0	0	0	3.3	100.8	8.9
	Colon	0	0	0	0	0	3.4	13	9.7
	Rectum	0	0	0	0	0	3.7	12.1	10.6
	Stomach	0	0	0	0	0	3.2	6	11.6
	Esophagus	0	0	0	0	0	1.7	10.6	18.4
Kidney	Urinary bladder	0	0	0	0	0	6.2	17	8.5
	Kidney	0	0	0	0	0	1.1	110.2	15.3
Male tissue	Epididymis	0	22.5	33.5	0	0	0	14.8	10.6
	Seminal vesicle	0	0	0	0	0	0	3	17.5
	Prostate	0	0	0	0	0	1.5	5.6	22.9
	Testis	54	9.5	16.9	29.1	34.9	0	5.4	28.9
Female tissue	Cervix, uterine	0	0	0	0	0	1.8	7.2	10.5
	Breast	0	0	0	0	0	2.5	5.9	11.1
	Ovary	0	0	0	0	4.2	0	3.1	14.9
	Fallopian tube	0	0	0	0	0	1.4	12.3	15.1
	Placenta	0	0	0	0	0	4	11.5	16.4
	Endometrium	0	0	0	0	0	1.1	5.9	29.6
Fat	Adipose tissue	0	0	0	0	0	2.2	10.8	8.9
Skin	Skin	0	0	0	0	0	0	7.5	11.6

Supplementary Figure 8: Top two panels show tissue/cell RNA-Seq expression summary of *CXorf21* using the GTEx dataset[6] and the FANTOM5 dataset (data available from the Human Protein Atlas[7]). Tissues arranged on X-axis by highest to lowest expression of *CXorf21*. GTEx Y-axis: RPKM, FANTOM5 Y-axis: TPM. Bottom panel shows RNA-Seq expression summary of *CXorf21* across a range of cell lines coloured by lineage. *CXorf21* expression is greatest in the THP-1 cell-line (myeloid). Data are available from the Human Protein Atlas.

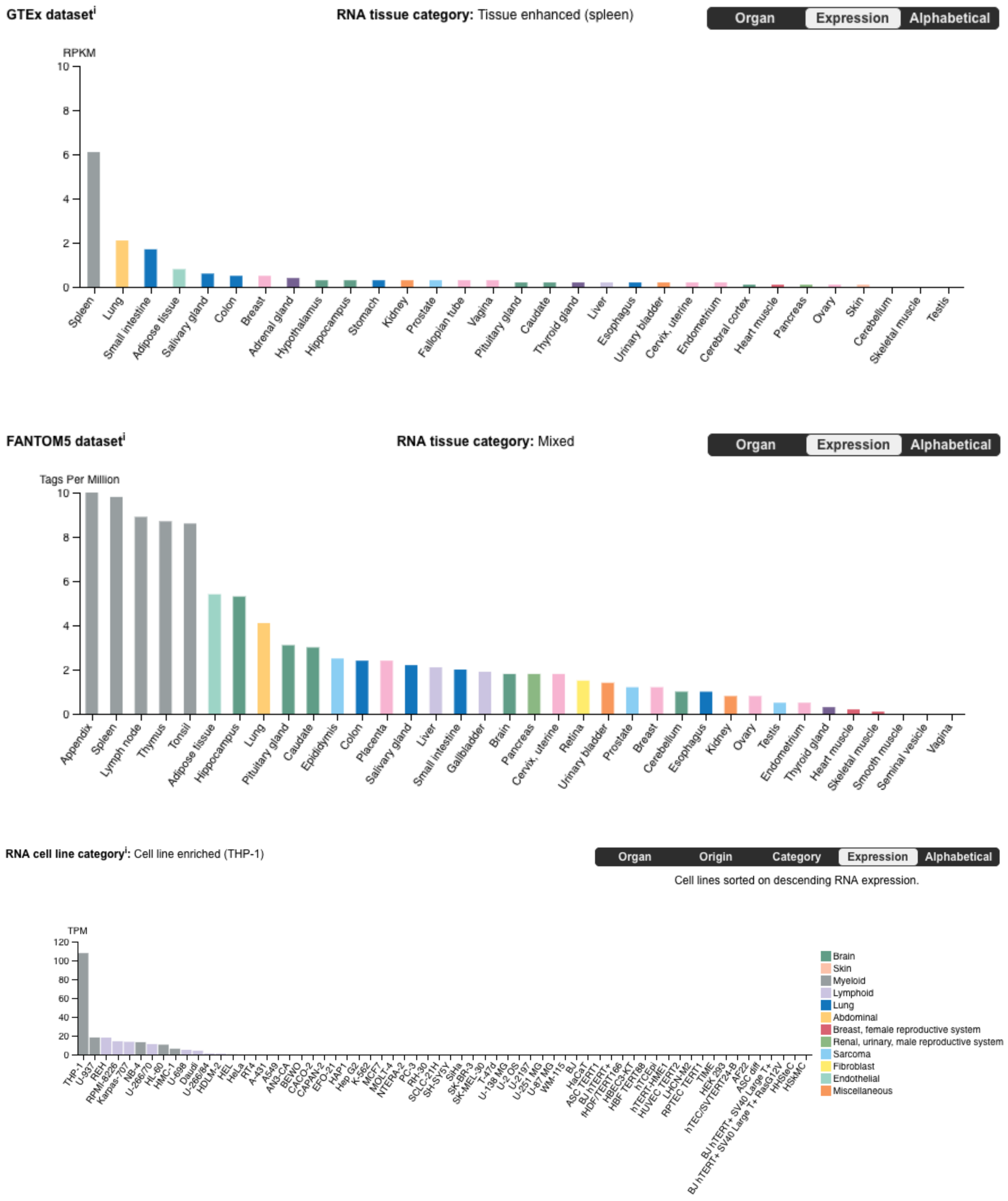

Supplementary Figure 9: Expression of *CXorf21* using data taken from primary immune cell-types using Blueprint-Epigenome[4] RNA-Seq (top-panel) reported as RPKM (Reads Per Kilobase of transcript per Million mapped reads) and in primary immune cell-types using BioGPS (probe ID: 220252_x_at) microarray (bottom-panel).

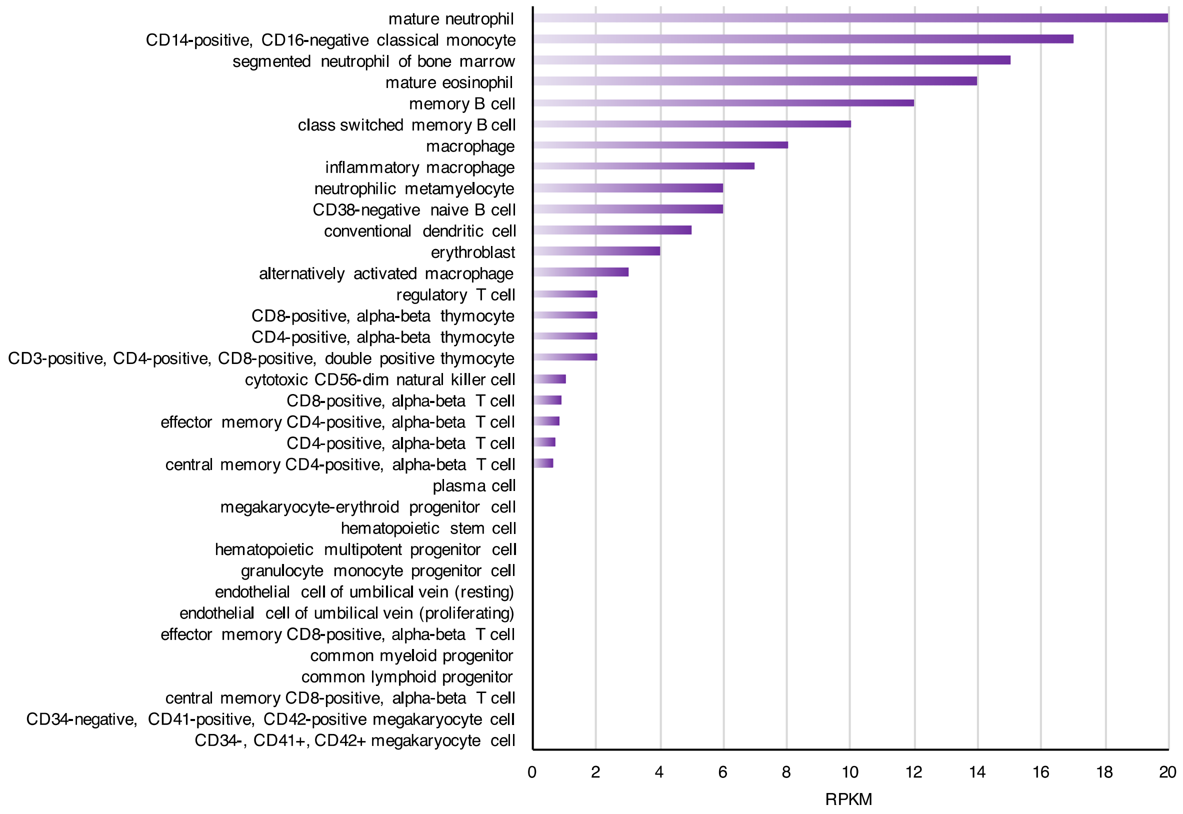

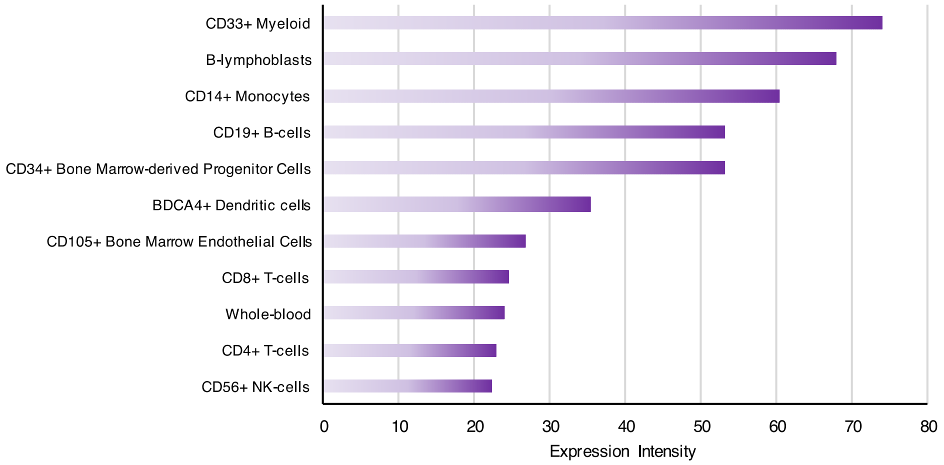

Supplementary Figure 10: Chromatin state model based on imputed data (25 state, 12 marks, 127 epigenomes) from the Roadmap Epigenomics Project[8] (A chromatin state model based on the imputed data for 12 marks, H3K4me1, H3K4me2, H3K4me3, H3K9ac, H3K27ac, H4K20me1, H3K79me2, H3K36me3, H3K9me3, H3K27me3, H2A.Z, and DNase, across all 127 reference epigenomes with 25-states was learned). Chromatin state for each gene in the 1Mb window surrounding the lead SLE associated SNP rs887369 (chrX: 30,077,846-31,077,845) is shown. Each panel corresponds to the length of each gene with the transcription start site (TSS) on the left of each box, and the transcription termination site (TTS) on the right of each box. The colours represent the state of the chromatin. The Roadmap Epigenomics categories for tissue/cell-type for 'Blood and T-cell' and 'haematopoietic stem cell (HSC) and B-cell' are highlighted to show the unique immune-specific chromatin landscape of the *CXorf21* promoter region.

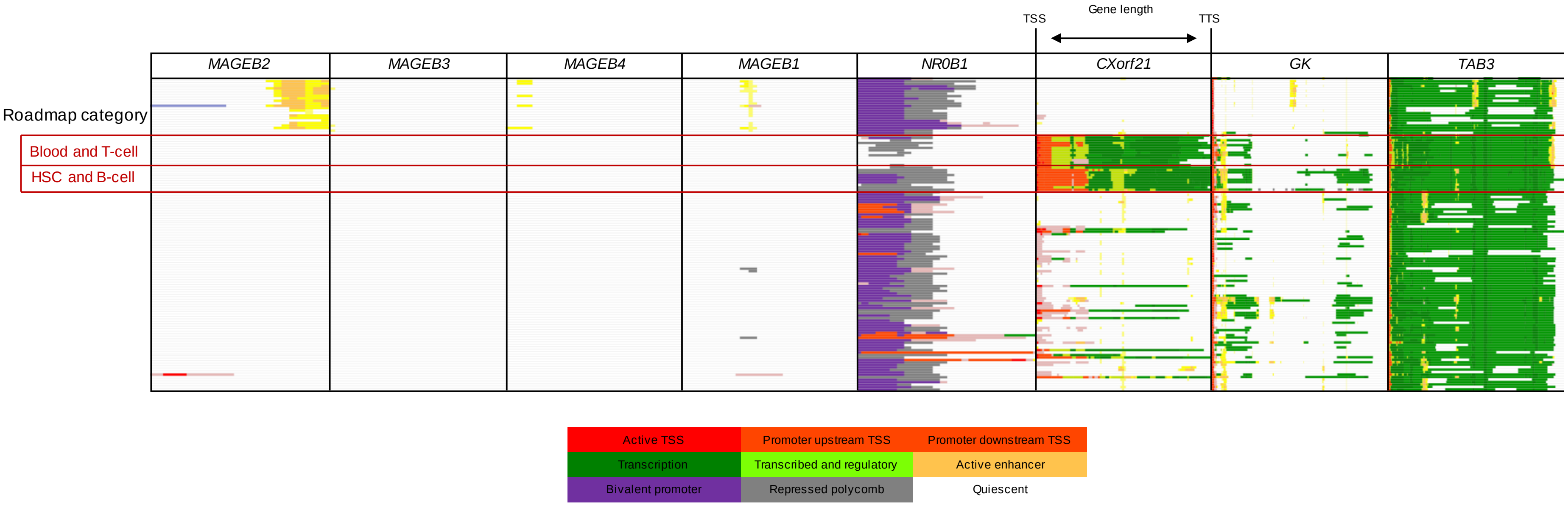

Supplementary Figure 11: Top panel: protein expression data is shown for CXORF21 for each of the 44 tissues assayed in the Human Protein Atlas[7]. Colour coding is based on tissue groups. Bottom panel: antibody validation of the CXORF21 antibody from the Human Protein Atlas by Western Blot across 3 cell lines. The same antibody validated by the Human Protein Atlas was used for the functional work in the manuscript (Atlas Antibodies Cat# HPA001185, RRID: AB_1078591).

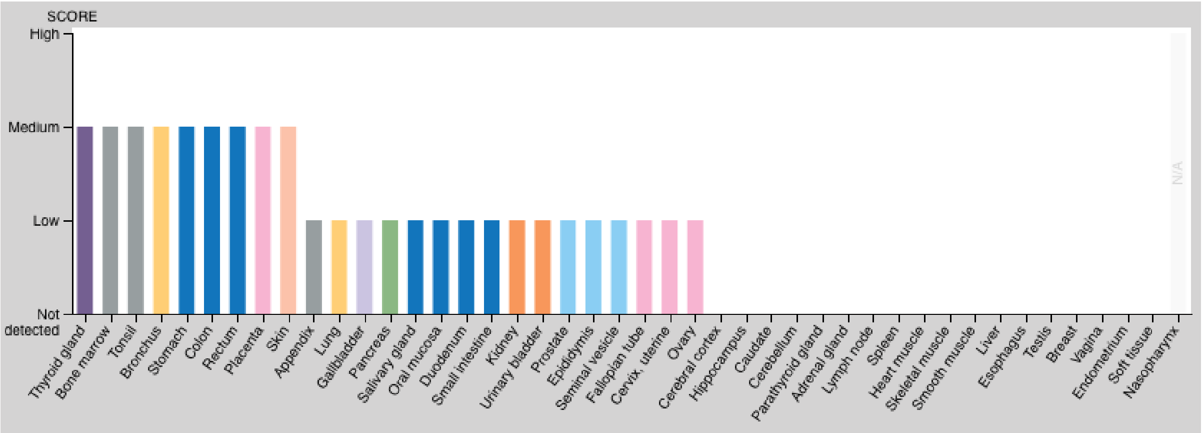

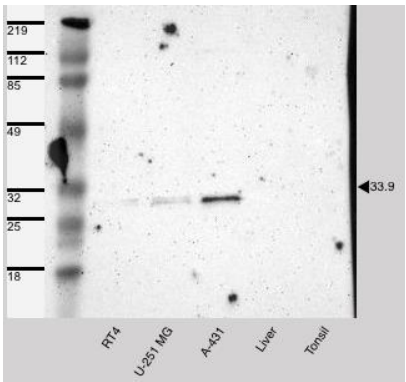

RT4: urinary bladder cancer cell line
U-251MG: brain cancer cell line
A-431: skin cancer cell line

Supplementary Figure 12: *Ex vivo* PBMCs from SLE cases and healthy controls were used to measure the expression of CXORF21 in both resting (n_{cases} = 19; $n_{controls}$ = 13) and IFN-stimulated (n_{cases} = 14; $n_{controls}$ = 13) monocytes and B cells. **a:** Gating strategy using FlowJo v.10.1 software. CD14+ and CD19+ cells, representing monocytes and B cells respectively, were identified by the positive peak in the histograms (middle panels). These populations are overlaid in the FSC/SSC representing their position within the PBMC population (upper panel). Representative mean fluorescent intensity (MFI) of CXORF21 and isotype control (IC) are shown in the lower panels. The CXORF21 MFI was calculated as $MFI_{CXORF21} – MFI_{IC}$. **b:** The means of CXORF21 MFI of cases and controls were compared for each of four cell types using an unpaired t-test. No significant difference between SLE cases and controls was observed in either cell type or condition following Bonferroni correction. Box-plots show minimum (Q1-1.5*IQR), 25th percentile (Q1), Median, 75th percentile (Q3), and maximum Q3+1.5*IQR. Source data are provided as a Source Data file.

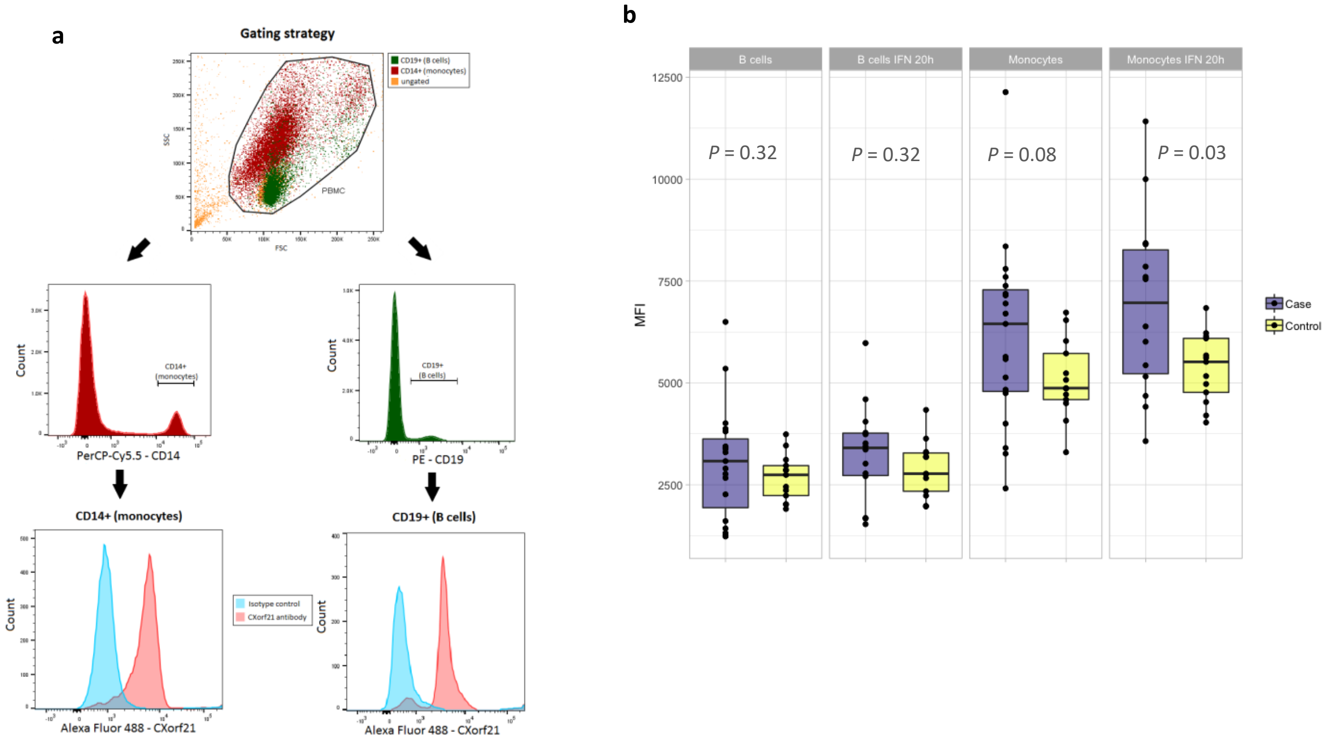

Supplementary Figure 13: *Ex vivo* PBMCs from SLE cases (n=19) were used to measure the expression of CXORF21 in fluorescently stained monocytes (CD14+) and B cells (CD19+). The mean fluorescent intensity (MFI) of CXORF21 was calculated as $MFI_{CXORF21} - MFI_{IC}$. (see Supplementary Figure 12). SLE Disease Activity Index (SLEDAI) was calculated for each patient at time of sampling. Regression models were fitted for CXORF21 as a function of SLEDAI as a single variable (upper panels) and CXORF21 as a function of SLEDAI stratified by age (lower panels). Source data are provided as a Source Data file.

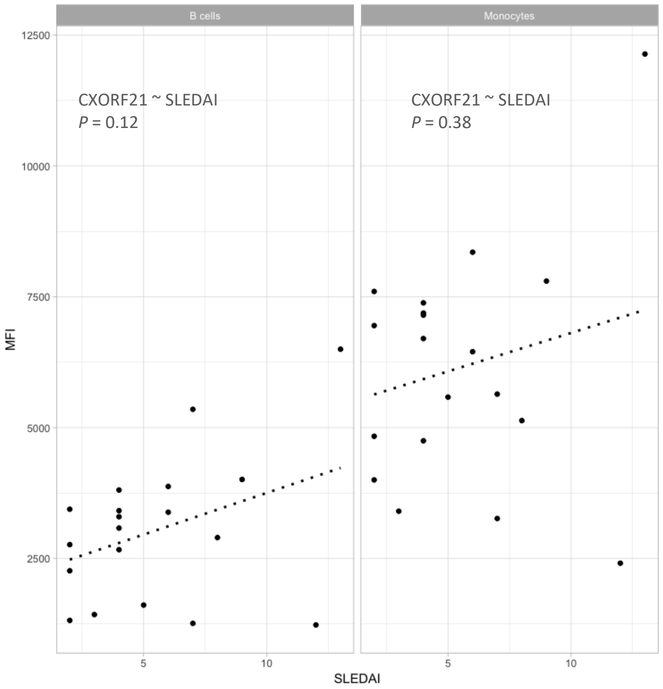

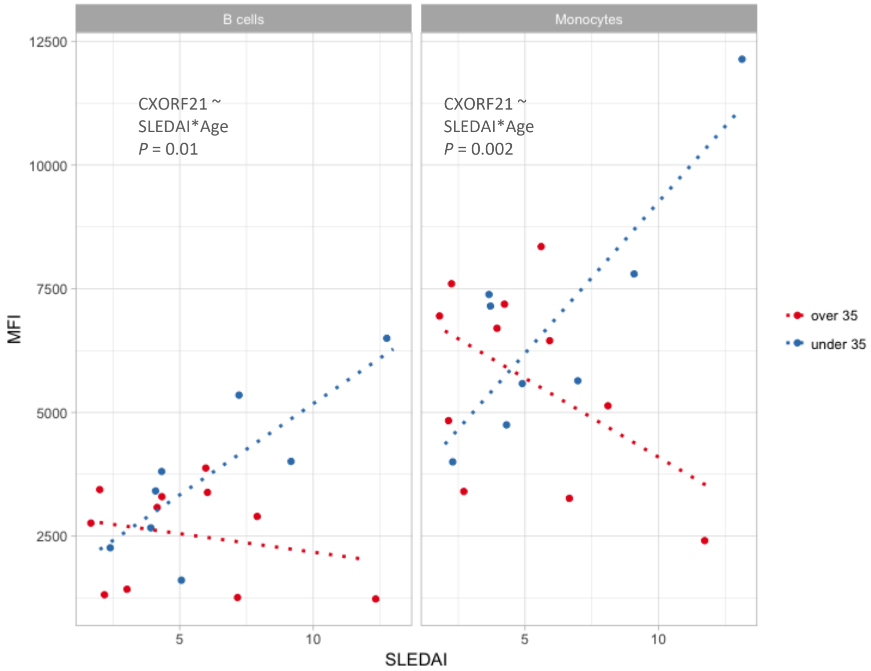

Supplementary Figure 14: Resting *ex vivo* PBMCs from healthy female controls (n = 3) were stained for CXORF21 (green) and either **a:** Golgi **b:** Nuclear or **c:** Lysosomal markers (see Methods). Using the co-localisation mask on the IDEAS software, no co-localisation (Bright Intensity Similarity) was observed with any of the three organelles in any sample. Representative data from one individual are shown. BF = Bright Field.

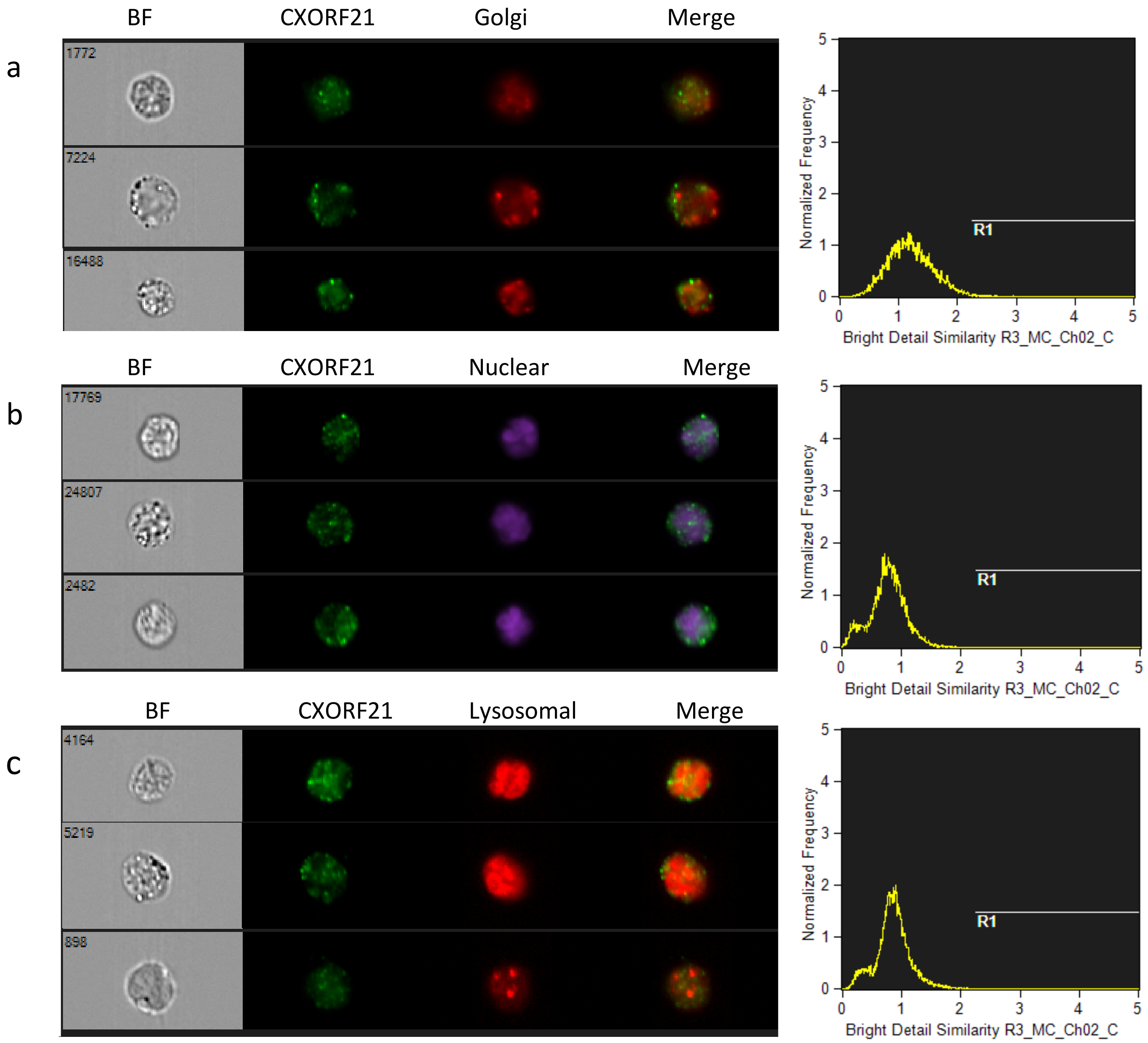

Supplementary Figure 15: Resting *ex vivo* PBMCs from healthy female controls (n = 3) were stimulated with IFN and stained for CXORF21 (green) and either **a:** Golgi **b:** Nuclear or **c:** Lysosomal markers (see methods). Using the co-localisation mask on the IDEAS software, no co-localisation (Bright Intensity Similarity) was observed with any of the three organelles in any sample. Representative data from one individual shown. BF = Bright Field.

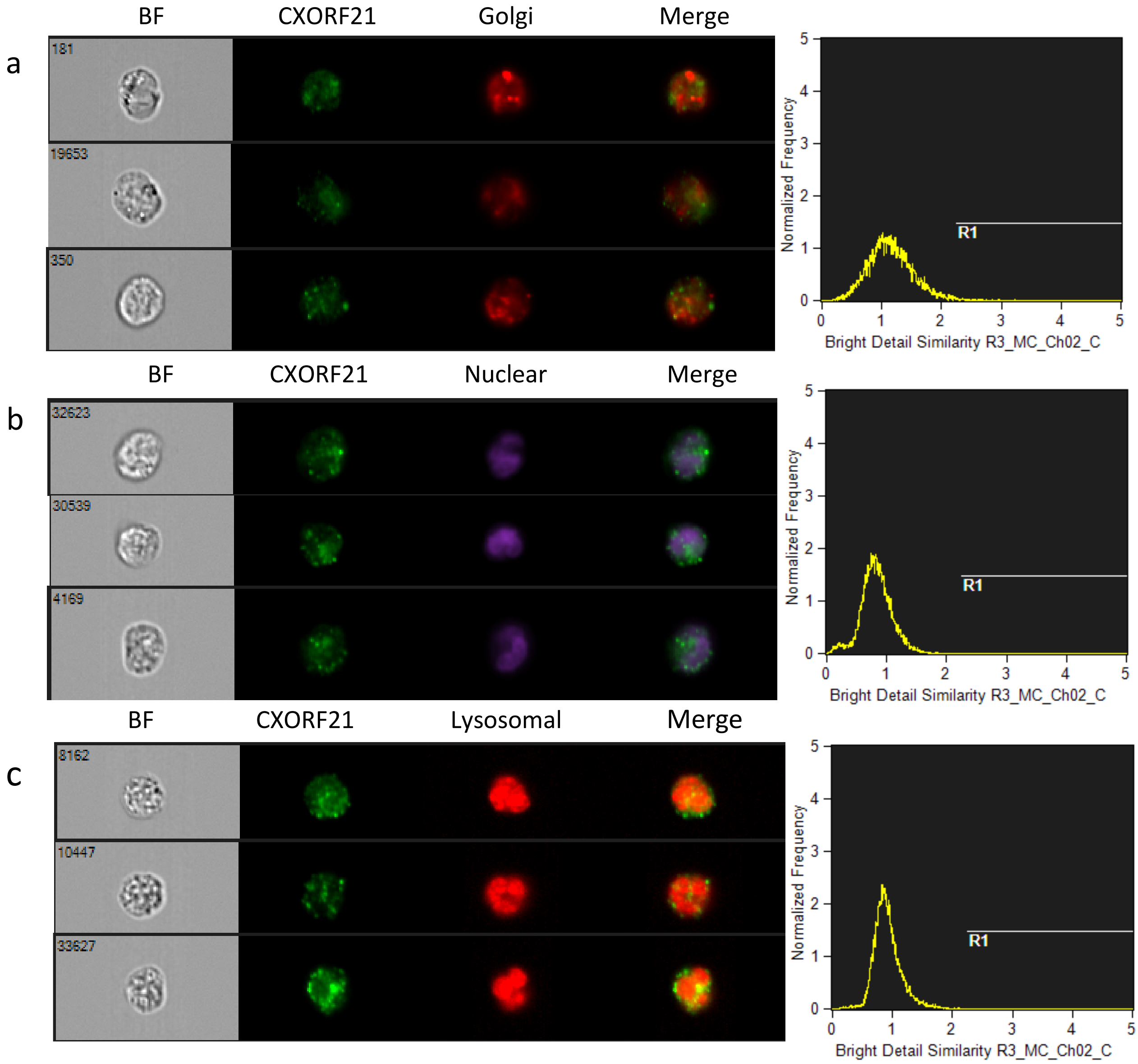

Supplementary Table 1: Differential H3K36me3 signal between the male and female samples across cell-types using the Blueprint Data[4]. Sample size per group.

Cell type	Female	Male
Alternatively activated macrophage	1	2
CD14-positive, CD16-negative classical monocyte	2	2
CD38-negative naive B cell	1	1
CD4-positive, alpha-beta T cell	1	2
central memory CD4-positive, alpha-beta T cell	0	1
class switched memory B cell	1	2
immature conventional dendritic cell	0	3
inflammatory macrophage	1	4
macrophage	2	3
mature conventional dendritic cell	0	3
mature eosinophil	1	1
mature neutrophil	2	10
monocyte	2	1
naive B cell	1	4
osteoclast	0	2
Un-switched memory B cell	0	1

Supplementary Table 2: Differential expression analysis of *CXorf21* between males and females using GTEx RNA-Seq[6] (TMP) data across the 45 cell/tissue types where expression data were available for both sexes. An unpaired *t*-test was performed between males and females after grouping by cell/tissue type. Significant results are highlighted which pass the Bonferroni corrected multiple testing cut-off of $P_{BF} < 0.05$.

cell_tissue_type	male_median_tpm	female_median_tpm	t_value	p_value	BF_adjusted_p_value
**Cells - EBV-transformed lymphocytes**	**37.45**	**64.565**	**-5.8031004**	**2.46E-07**	**1.10E-05**
**Thyroid**	**0.5567**	**0.7385**	**-4.108077**	**5.88E-05**	**0.00264731**
Adipose - Visceral (Omentum)	2.069	1.504	2.36493057	0.0187434	0.84345299
Whole Blood	12.22	16.485	-1.9611539	0.05077572	1
Esophagus - Mucosa	0.48745	0.4302	1.82464908	0.06879074	1
Skin - Not Sun Exposed (Suprapubic)	0.3751	0.4611	-1.6199539	0.10651406	1
Adipose - Subcutaneous	1.424	1.782	-1.5823361	0.11458703	1
Cells - Transformed fibroblasts	0	0	1.57989654	0.11531565	1
Heart - Atrial Appendage	0.2825	0.2624	1.50305059	0.13392554	1
Lung	5.1995	5.471	-1.4318349	0.1537022	1
Brain - Cerebellar Hemisphere	0.1302	0.1445	1.25259382	0.2125352	1
Brain - Caudate (basal ganglia)	0.3946	0.3608	1.24294107	0.21614253	1
Liver	0.28755	0.425	-1.2313997	0.22088709	1
Esophagus - Muscularis	0.4203	0.3971	-1.135761	0.25729294	1
Kidney - Cortex	0.42645	0.5316	-1.1587325	0.27216404	1
Nerve - Tibial	1.4425	1.362	-1.0958559	0.27427789	1
Pancreas	0.2022	0.1387	1.04045358	0.29956074	1
Brain - Cortex	0.24545	0.25735	1.02939316	0.30499083	1
Artery - Coronary	1.713	1.197	1.01341083	0.31238319	1
Breast - Mammary Tissue	0.8772	1.194	-1.0000558	0.31818071	1
Colon - Transverse	1.12	1.0695	0.94518654	0.34553226	1
Brain - Frontal Cortex (BA9)	0.21525	0.3796	-0.919433	0.36206095	1
Colon - Sigmoid	0.61645	0.6369	0.81633733	0.41521005	1
Brain - Cerebellum	0.09479	0.1478	-0.6867748	0.49343945	1
Esophagus - Gastroesophageal Junction	0.4278	0.4469	-0.6284226	0.53068871	1
Heart - Left Ventricle	0.14525	0.1545	-0.5922672	0.55421663	1
Minor Salivary Gland	1.207	1.0109	0.52969396	0.59852114	1
Small Intestine - Terminal Ileum	2.014	3.381	-0.510399	0.61074932	1
Skin - Sun Exposed (Lower leg)	0.3462	0.3706	-0.5083253	0.61157481	1
Bladder	0.8298	0.2907	0.52591862	0.61257721	1
Spleen	14.185	14.385	-0.4209835	0.67438039	1
Brain - Putamen (basal ganglia)	0.2208	0.3163	-0.3186297	0.75071581	1
Brain - Substantia nigra	0.7486	0.6533	0.28560185	0.77602033	1
Brain - Hypothalamus	0.5517	0.45055	-0.2600743	0.79569233	1
Artery - Tibial	0.6778	0.6617	0.25121458	0.80181913	1
Adrenal Gland	0.9439	0.85595	-0.2148127	0.83023299	1
Artery - Aorta	1.1365	1.381	-0.1746123	0.86151891	1
Stomach	0.64445	0.55395	-0.1507854	0.88032744	1
Pituitary	0.4777	0.4761	0.14338865	0.88627962	1
Brain - Nucleus accumbens (basal ganglia)	0.3154	0.2881	-0.0655375	0.94793057	1
Muscle - Skeletal	0.06088	0.06271	-0.0590894	0.95290557	1
Brain - Hippocampus	0.4088	0.38445	-0.0507728	0.9596361	1
Brain - Amygdala	0.401	0.50395	0.03655186	0.97092271	1
Brain - Spinal cord (cervical c-1)	2.0255	1.493	0.008136	0.99353493	1
Brain - Anterior cingulate cortex (BA24)	0.24195	0.2282	0.00619426	0.99507028	1

Supplementary Table 3: Expression profile of *CXorf21* in primary *ex vivo* cell types between male and female individuals using separate studies by *Fairfax et al*[2] and *Naranbhai*[3] *et al* (see methods). *CXorf21* expression is measured by microarray (probe ID: ILMN_1651752). An unpaired *t*-test was performed between males and females after grouping by cell/tissue type. Significant results are highlighted which pass the Bonferroni corrected multiple testing cut-off of $P_{BF} < 0.05$.

cell_tissue_type	male_mean_tpm	female_mean_tpm	male_sample_size	female_sample_size	t_value	p_value	BF_adjusted_p_value
**Monocyte LPS24 2640397**	**7.96008345**	**8.22491473**	**153**	**169**	**-7.3765748**	**1.41E-12**	**9.84E-12**
**Monocyte IFN-G 24h 2640397**	**8.02417359**	**8.19649517**	**173**	**194**	**-5.450372**	**9.29E-08**	**6.50E-07**
Monocyte 2640397	7.76114056	7.82406513	186	228	-2.240201	0.02562403	0.17936822
Neutrophil 2640397	7.87952502	7.91085788	51	50	-0.9213874	0.35909297	1
NK 2640397	7.19056865	7.19548385	118	127	-0.8436036	0.39972074	1
Monocyte LPS2 2640397	7.43098103	7.4069583	130	131	0.61622389	0.5382883	1
B-cell 2640397	7.1694935	7.18024787	121	159	-0.545171	0.58607751	1

Supplementary Table 4: Functional characterization of the eight genes within the 1Mb locus of lead SLE associated SNP rs887369.

Gene	Description	GTEx Tissues (RPKM > 5)	Human protein Atlas (TPM > 10)	OMIM	PUBMED (Gene name AND SLE)	BioPlex Protein Interactions	Entrez Description
MAGEB2	Melanoma antigen family B2	Testis (20.876)	Testis (54)	NA	MAGE Xp-2: a member of the MAGE gene family isolated from an expression library using systemic lupus erythematosus sera; MAGE-B2 autoantibody: a new biomarker for paediatric systemic lupus erythematosus.	NA	This gene is a member of the MAGEB gene family. The members of this family have their entire coding sequences located in the last exon, and the encoded proteins show 50 to 68% sequence identity to each other. The promoters and first exons of the MAGEB genes show considerable variability, suggesting that the existence of this gene family enables the same function to be expressed under different transcriptional controls. This gene is localized in the DSS (dosage-sensitive sex reversal) critical region. It is expressed in testis and placenta, and in a significant fraction of tumours of various histological types. The MAGEB genes are clustered on chromosome Xp22-p21.
MAGEB3	Melanoma antigen family B3	NA	Epididymis (22.5)<br>Testis (9.5)	NA	NA	MAGEA1, MAGEB3, PPP1R7	As above.
MAGEB4	Melanoma antigen family B4	Testis (6.271)	Epididymis (33)<br>Testis (16.9)	NA	NA	MAGEB10, MAGEB4, MAGEA1, C1QBP, CAMK2D, ROCK1	As above.
MAGEB1	Melanoma antigen family B1	Testis (12.801)	Testis (29.1)	NA	NA	NA	As above.
NR0B1	Nuclear receptor subfamily 0, group B, member 1	Adrenal gland (12.853)<br>Testis (16.157)	Adrenal gland (34.9)<br>Testis (27.9)	46XY sex reversal 2, dosage-sensitive; Adrenal hypoplasia, congenital	NA	NA	This gene encodes a protein that contains a DNA-binding domain. The encoded protein acts as a dominant-negative regulator of transcription which is mediated by the retinoic acid receptor. This protein also functions as an anti-testis gene by acting antagonistically to Sry. Mutations in this gene result in both X-linked congenital adrenal hypoplasia and hypogonadotropic hypogonadism.
CXorf21	Chromosome X open reading frame 21	LCLs (18.496)<br>Whole-blood (6.686)<br>Spleen (6.123)	Appendix (14.8)<br>Spleen (13.4)<br>Bone Marrow (12)<br>Lymph Node (11.7)<br>Tonsil (9.4)	NA	NA	SLC15A4	NA
GK	Glycerol kinase	Whole-blood (13.925)<br>Liver (8.339)<br>Lung (6.113)	Kidney (110.2)<br>Small Intestine (100.8)<br>Duodenum (89.7)<br>Liver (41.6)	Glycerol kinase deficiency	NA	CD70, FCGR3A, GHITM, FPR2, SYP, IL13RA2, APLNR, VSIG4, TNF, GK, GK3P, UBP1, AZGP1, SCGB1D2, APOD, HBB, VSIG8, MUC20, SLC22A6, RSPRY1, SLC2A12, SLC5A8, PMEL, PDCD1, VSIG1, MFSD4, CHRM4, PTGIR	The protein encoded by this gene belongs to the FGGY kinase family. This protein is a key enzyme in the regulation of glycerol uptake and metabolism. It catalyses the phosphorylation of glycerol by ATP, yielding ADP and glycerol-3-phosphate. Mutations in this gene are associated with glycerol kinase deficiency (GKD). Alternatively-spliced transcript variants encoding different isoforms have been found for this gene.
TAB3	TGF-beta activated kinase 1/MAP3K7 binding protein 3	Uterus (7.661)<br>Testis (6.037)	Parathyroid gland (52.6)<br>Endometrium (29.6)<br>Testis, Prostate (28.9)	NA	NA	EMILIN1	The product of this gene functions in the NF-kappa B signal transduction pathway. The encoded protein, and the similar and functionally redundant protein MAP3K7IP2/TAB2, forms a ternary complex with the protein kinase MAP3K7/TAK1 and either TRAF2 or TRAF6 in response to stimulation with the pro-inflammatory cytokines TNF or IL-1. Subsequent MAP3K7/TAK1 kinase activity triggers a signalling cascade leading to activation of the NF-kappa B transcription factor. The human genome contains a related pseudogene. Alternatively-spliced transcript variants have been described, but their biological validity has not been determined.

Annotation of genes within 1Mb of rs887369 (chrX: 30,077,846-31,077,845). Gene name and description taken from Entrez. For GTEx expression[6], the three most expressed cell/tissue types are reported that have an RPKM greater than 5. For Human Protein Atlas Expression[7], again the three most significantly expressed cell / tissue types are reported and expression is considered for genes with a TPM greater than 10. OMIM: Gene-Phenotype Relationships are reported for each gene. A PubMed search was conducted using key words: ‘gene name AND SLE’ as well as ‘gene name AND lupus’ and the publications listed. BioPlex 2.0 Version 4[9] was interrogated for protein-protein interactions in human cell lines and each interaction reported using the Core Sub-Network Protein Interaction Information table. GTEx: Genotype-Tissue Expression project; RPKM: Reads Per Kilobase of transcript per Million; TPM: Transcripts Per Million; OMIM: Online Mendelian Inheritance in Man; BioPlex: biophysical interactions of ORFeome-based complexes.

Supplementary Table 5: Gene function estimation of *CXorf21* by gene expression co-regulation profiling across a multitude of cell and tissue types using COXPRESdb[10].

Gene	Function	Hsa3 MR for CXorf21
CXorf21	chromosome X open reading frame 21	0
GPR65	G protein-coupled receptor 65	2.8
LAT2	linker for activation of T cells family, member 2	3.5
LILRB1	leukocyte immunoglobulin-like receptor, subfamily B (with TM and ITIM domains), member 1	3.7
TLR7	toll-like receptor 7	4.5
DAPP1	dual adaptor of phosphotyrosine and 3-phosphoinositides	7.2
TLR6	toll-like receptor 6	8.7
ARL11	ADP-ribosylation factor-like 11	9.4
TLR10	toll-like receptor 10	10.1
ST8SIA4	ST8 alpha-N-acetyl-neuraminide alpha-2,8-sialyltransferase 4	10.2
CARD8-AS1	CARD8 antisense RNA 1	10.3
IPCEF1	interaction protein for cytohesin exchange factors 1	10.9
SNX20	sorting nexin 20	11.3
ITGAL	integrin, alpha L (antigen CD11A (p180), lymphocyte function-associated antigen 1; alpha polypeptide)	12.2
KLHL6	kelch-like family member 6	12.7
PLAC8	placenta-specific 8	13.1
GVINP1	GTPase, very large interferon inducible pseudogene 1	15
CD84	CD84 molecule	15.6
PIK3CD-AS1	PIK3CD antisense RNA 1	16.9
CIITA	class II, major histocompatibility complex, transactivator	17.8
SLAMF7	SLAM family member 7	18.3
SLAMF6	SLAM family member 6	18.6
PTPLAD2	protein tyrosine phosphatase-like A domain containing 2	19.9
LAX1	lymphocyte transmembrane adaptor 1	20.8
SCIMP	SLP adaptor and CSK interacting membrane protein	21.9
LINC00926	long intergenic non-protein coding RNA 926	22.4
PCED1B-AS1	PCED1B antisense RNA 1	24.7
POU2AF1	POU class 2 associating factor 1	24.8
SPN	sialophorin	25.3
IL12RB1	interleukin 12 receptor, beta 1	25.5
P2RY10	purinergic receptor P2Y, G-protein coupled, 10	26.5
LOC101928429	uncharacterized LOC101928429	27.5
ZBP1	Z-DNA binding protein 1	29.6
LOC101929889	leukosialin-like	29.7
STX11	syntaxin 11	30.3
TMEM156	transmembrane protein 156	30.6
NLRP3	NLR family, pyrin domain containing 3	30.6
PTAFR	platelet-activating factor receptor	31.4
NFAM1	NFAT activating protein with ITAM motif 1	31.4
LY75	lymphocyte antigen 75	32.2
IRAK3	interleukin-1 receptor-associated kinase 3	33.6
GAPT	GRB2-binding adaptor protein, transmembrane	37.8
MS4A7	membrane-spanning 4-domains, subfamily A, member 7	40.3
CTSS	cathepsin S	40.9
LINC00528	long intergenic non-protein coding RNA 528	41.4
FFAR2	free fatty acid receptor 2	41.5

FGD2	FYVE, RhoGEF and PH domain containing 2	42.1
LRRC25	leucine rich repeat containing 25	42.7
SIGLEC14	sialic acid binding Ig-like lectin 14	42.7
LAIR1	leukocyte-associated immunoglobulin-like receptor 1	43.4
GPR18	G protein-coupled receptor 18	44.2
HSH2D	hematopoietic SH2 domain containing	45.2
GPR132	G protein-coupled receptor 132	45.3
CR1	complement component (3b/4b) receptor 1 (Knops blood group)	46.3
GPR174	G protein-coupled receptor 174	47
PARVG	parvin, gamma	49.1
LOC101927069	uncharacterized LOC101927069	49.7
FCN1	ficolin (collagen/fibrinogen domain containing) 1	50
GBP4	guanylate binding protein 4	50.8
JAK3	Janus kinase 3	51
CLEC17A	C-type lectin domain family 17, member A	51.4
KCNA3	potassium voltage-gated channel, shaker-related subfamily, member 3	51.6
CLEC7A	C-type lectin domain family 7, member A	51.6
LOC729683	uncharacterized LOC729683	52
C16orf54	chromosome 16 open reading frame 54	52.8
CD96	CD96 molecule	58.7
LINC00996	long intergenic non-protein coding RNA 996	59.6
IL21R	interleukin 21 receptor	60.2
STK4	serine/threonine kinase 4	60.7
SFMBT2	Scm-like with four mbt domains 2	61.1
LILRA5	leukocyte immunoglobulin-like receptor, subfamily A (with TM domain), member 5	61.4
HLA-DQB2	major histocompatibility complex, class II, DQ beta 2	64.6
SIGLEC10	sialic acid binding Ig-like lectin 10	65
CECR1	cat eye syndrome chromosome region, candidate 1	65.9
HAVCR2	hepatitis A virus cellular receptor 2	67.5
GBP5	guanylate binding protein 5	68.8
TRG-AS1	T cell receptor gamma locus antisense RNA 1	69.3
CLECL1	C-type lectin-like 1	70
NLRC3	NLR family, CARD domain containing 3	70.4
IRF4	interferon regulatory factor 4	72.4
MAP4K1	mitogen-activated protein kinase kinase kinase kinase 1	74.2
ENTPD1	ectonucleoside triphosphate diphosphohydrolase 1	76.3
PTPRC	protein tyrosine phosphatase, receptor type, C	77.7
CRLF3	cytokine receptor-like factor 3	78.8
IL2RA	interleukin 2 receptor, alpha	80.1
CCR6	chemokine (C-C motif) receptor 6	81
CASP10	caspase 10, apoptosis-related cysteine peptidase	81.1
NCF1B	neutrophil cytosolic factor 1B pseudogene	81.2
CCL22	chemokine (C-C motif) ligand 22	83.1
ZC3H12D	zinc finger CCCH-type containing 12D	83.2
FUT4	fucosyltransferase 4 (alpha (1,3) fucosyltransferase, myeloid-specific)	87.5
MILR1	mast cell immunoglobulin-like receptor 1	88.3
SYK	spleen tyrosine kinase	89.5
LILRB3	leukocyte immunoglobulin-like receptor, subfamily B (with TM and ITIM domains), member 3	89.5
CD300LB	CD300 molecule-like family member b	90.5
TNFSF14	tumor necrosis factor (ligand) superfamily, member 14	90.5
SERPINB9	serpin peptidase inhibitor, clade B (ovalbumin), member 9	90.8
STAP1	signal transducing adaptor family member 1	91

SIRPB2	signal-regulatory protein beta 2	91.2
CLEC4E	C-type lectin domain family 4, member E	91.6
FCGR2C	Fc fragment of IgG, low affinity IIc, receptor for (CD32) (gene/pseudogene)	91.9
SASH3	SAM and SH3 domain containing 3	94
TESPA1	thymocyte expressed, positive selection associated 1	94
P2RY8	purinergic receptor P2Y, G-protein coupled, 8	94.1
NCF1C	neutrophil cytosolic factor 1C pseudogene	95.3
HLA-DOA	major histocompatibility complex, class II, DO alpha	95.5
FCRL1	Fc receptor-like 1	95.8
PIK3CG	phosphatidylinositol-4,5-bisphosphate 3-kinase, catalytic subunit gamma	97.2
PIK3CD	phosphatidylinositol-4,5-bisphosphate 3-kinase, catalytic subunit delta	97.5
CARD8	caspase recruitment domain family, member 8	98
PHF11	PHD finger protein 11	98
TREML2	triggering receptor expressed on myeloid cells-like 2	99.8

Performed using the COXPRESdb version 6.0 algorithm. *CXorf21* was used as the query. The co-expressed genes with an MR (mutual rank below 100 are reported; calculated as an average of the rank of gene B in the co-expressed genes to gene A and the average of the rank of gene A to gene B) are reported for Hsa3 (homo sapiens) genes using expression data profiled using RNA-Sequencing (see publication). The MR value is reported for each co-expressed gene. Only genes with two or three starts (level of supportability) are used in analysis (see publication). The genes implicated in the KEGG toll-like receptor signalling pathway are highlighted in bold.

Supplementary References

1. Lappalainen, T. *et al.* Transcriptome and genome sequencing uncovers functional variation in humans. *Nature* **501,** 506–11 (2013).
2. Fairfax, B. P. *et al.* Innate immune activity conditions the effect of regulatory variants upon monocyte gene expression. *Science* **343,** 1246949 (2014).
3. Naranbhai, V. *et al.* Genomic modulators of gene expression in human neutrophils. *Nat. Commun.* **6,** 7545 (2015).
4. Martens, J. H. A. & Stunnenberg, H. G. BLUEPRINT: Mapping human blood cell epigenomes. *Haematologica* **98,** 1487–1489 (2013).
5. The ENCODE Project Consortium *et al.* An integrated encyclopedia of DNA elements in the human genome. *Nature* **489,** 57–74 (2012).
6. The GTEx Consortium *et al.* The Genotype-Tissue Expression (GTEx) project. *Nat. Genet.* **45,** 580–585 (2013).
7. Pont´en, F., Jirstr¨om, F. & Uhlen, M. The Human Protein Atlas — a tool for pathology. *J. Pathol.* **216,** 387–393 (2008).
8. Roadmap Epigenomics Consortium *et al.* Integrative analysis of 111 reference human epigenomes. *Nature* **518,** 317–330 (2015).
9. Huttlin, E. L. *et al.* The BioPlex Network: A Systematic Exploration of the Human Interactome. *Cell* **162,** 425–440 (2015).
10. Okamura, Y. *et al.* COXPRESdb in 2015 : coexpression database for animal species by DNA-microarray and RNAseq-based expression data with multiple quality assessment systems. **43,** 82–86 (2015).
